# The Plea of Insanity in Criminal Cases
*Croonian Lectures on Medical Testimony and Evidence in Cases of Lunacy. By Thomas Mayo, M.D., "Medical Times and Gazette," Nos. 180, 181, 182.Unsoundness of Mind considered in Relation to the Question of Responsibility for Criminal Acts. By Samuel Knaggs. London: Churchill. 1854.


**Published:** 1854-04-01

**Authors:** 


### Art. II.—THE PLEA OF INSANITY IN CRIMINAL CASES.*

In the History of Medical Jurisprudence, we find no chapter more perplexing than that which regards the plea of insanity in criminal cases. Our courts of justice, whether civil or criminal, demand that the evidence shall on all occasions, be clear, conclusive, and indisputable. But, unhappily, the human mind, when affected by disease, cannot in every case have its morbid features unveiled in open court. When the plea of insanity, therefore, is raised, there is often considerable difficulty in bringing forward a sufficient amount of demonstrative proof to satisfy the minds of unprofessional men, that the malady actually exists. The law presumes every man to be in his sound senses—and, therefore, responsible for his actions, until the contrary be shown. It is important for the interests of society, that the ends of justice should not be evaded by any fictitious plea. Hence the judges of the land always receive the plea of insanity with great caution; and lawyers accustomed to deal, for the most part, with demonstrative evidence, listen with impatience to medical testimony, which cannot be reduced to the same description of proof which they require to establish ordinary matters of fact. "We want *facts*," they exclaim, "not opinions;" but the truth is, the most self-evident facts of medical science can only be viewed through the medium of opinion, which tact, knowledge, and experience bring within the range of certainty. If a man be suffering under some obscure organic disease of the heart or lungs, the prognosis of the physician is the expression of his opinion: but at the same time, his opinion carries with it—provided he be regarded as an authority—as much weight as if he could demonstrate the certainty

* Croonian Lectures on Medical Testimony and Evidence in Cases of Lunacy. By Thomas Mayo, M.D., "Medical Times and Gazette," Nos. 180, 181, 182.

Unsoundness of Mind considered in Relation to the Question of Responsibility for Criminal Acts. By Samuel Knaggs. London: Churchill. 1854.

of the impending fact with mathematical precision. Why then should the opinions of those medical men who have made the study of insanity a speciality, not be received as valid evidence? If a man, through the negligence of a public servant, or a railway company, meet with a severe bodily injury, for which he seeks compensation in a court of justice, the most eminent physicians and surgeons are examined, and their opinions are received as valid evidence concerning the nature, extent, and consequences of the injuries inflicted. When, however, the plea of insanity is at issue, their evidence is very often received with marked disrespect;—nay, not very long ago, upon the trial of Oxford, the Lord Chief Justice Denman is reported to have said:—

"There might be cases in which medical evidence as to physical symptoms was of the utmost consequence; but as to moral insanity, he, for his own part, could not admit that medical men had at all more means of forming an opinion on a case, than were possessed by gentlemen accustomed to the affairs of life, and bringing to the subject a wide experience."

This was equivalent to declaring that the opinions of Pinel, Esquirol, and Prichard, upon the difficult and perplexing subject of moral insanity, might any day be superseded by that of half-a-dozen members of the Carlton Club.

The directions which the learned judges have given to juries, in summing up the evidence of different state trials, indicate a fervent desire to lean to the side of humanity—but a great difficulty in determining what amount of insanity should render a man irresponsible for his actions. It is now almost inconceivable that so humane and enlightened a judge as Lord Chief Justice Hale, should, in drawing a distinction between total and partial insanity, have laid it down as the law that prisoners should be acquitted only in cases where a total and permanent want of reason was proved to exist. Under the head of total insanity, he distinguished between "that species which is fixed and permanent and lunacy which comes by periods or fits." It would appear that the superstition of a lunatic becoming deranged through the influence of the moon—whence the ignorant derivation of the word lunatic—at that time prevailed in the minds even of learned men; hence, when the moon was declining into its third quarter, there was supposed to be a remission of the insane symptoms, followed by a lucid interval.

"Crimes committed," says Judge Hale, "by lunatics, in such their distempers, are under the same judgment as those committed by men partially insane. The person who is absolutely mad for a day, killing a man in that distemper, is equally not guilty as if he were mad without intermission. But such persons as have their lucid intervals have usually in those intervals at least a competent use of reason; and crimes committed by them are of the same nature, and punished in the same manner, as if they had no such defect."

Hence arose another difficuly. What constitutes a lucid interval? How are we to measure its completeness or its duration? There may be a cessation of symptoms; the lunatic may become tranquil, and appear to act reasonably: there may be a calm on the surface of the waters, while below, the current may be flowing as disturbed as ever, although unobserved. Who can determine whether this state of mental tranquillity be real, profound, and enduring, or only, perhaps,an apparent lull, a hush, an intermission of the storm? And, above all, how can any human sagacity penetrate, during this so-called lucid interval, into the secret depths of the mind, in order to ascertain whether the motives—the actual springs of action—are perfectly sane, or perverted by silent delusions? It is comparatively easy to establish the existence of insanity,—but very difficult, if indeed it be even possible, to discover the precise time when the mind casts off the cloud which over-shadowed it, and recovers its perfect serenity. In the case of Arnold, who was indicted at Kingston-upon-Thames, before Mr. Justice Tracey, in the year 1724, for felony, in wilfully shooting at and wounding Lord Onslow, it was clearly shown that the man had been habitually insane; but because he had formed a regular design, and prepared the proper means for carrying his object into execution, and had, upon the morning of the day he committed the offence, acted with apparent rationality—inasmuch as he was capable of distinguishing the sort of shot he wanted for the purpose, which was larger than the ordinary size—it was contended that the act was perpetrated during a lucid interval; and the jury found him guilty. His insanity was so clearly proved, that his brothers and sisters were, in the course of the trial, severely censured for not having taken care of him, and for not adopting means for his being cured. It was shown that he had not only been long subjected to aural and visual illusions, but he was habitually under a variety of delusions; imagining, among other extravagances, that Lord Onslow was in his bosom, constantly persecuting him, and preventing him from eating, drinking, sleeping, or being at rest. But notwithstanding all this, the lucid interval was presumed to have been established; and Mr. Justice Tracey, in charging the jury, laid it down as the law, that a man to be exempted from punishment under the plea of insanity, "must be a man that is totally deprived of his understanding and memory, and does not know what he is doing any more than an infant, than a brute, or a wild beast."*

* State Trials, vol. xvi. Trial of Edward Arnold for Shooting at Lord Onslow, p. 766.

Upon the trial of James Hadfield, for high treason, in the year 1800, the Hon. Thomas Erskine, afterwards the renowned Lord Chancellor, in a speech remarkable alike for force of reasoning and beauty of language, analyzed and exposed the fallacy of the views which had been propounded both by Chief Justice Hale, and Mr. Justice Tracey; indeed, no medico-legal authority, enlightened by the most recent views of mental pathology, could explain in more clear and precise terms the true psychological principles which should guide our diagnosis in such cases.

"If" (said Lord Erskine) "a total deprivation of memory was intended by these great lawyers to be taken in the literal sense of the word—if it was meant that to protect a man from punishment, he must be in such a state of prostrated intellect as not to know his name, nor his condition, nor his relation towards others—that if a husband, he should not know he was married; or if a father, could not remember he had children; nor know the road to his house or his property in it, then no such madness ever existed in this world."

He then proceeded to argue truly, that,

"It is idiocy alone which places a man in this helpless condition, where, from an original mal-organization there is the human frame alone, without the human capacity, and which indeed meets the very definition of Lord Hale himself, when referring to Fitzherbert, he says, 'Idiocy, or fatuity, *à nativitate vel dementia naturalis*, is such a one as described by Fitzherbert: who knows not how to tell twenty shillings, nor knows his own age, or who was his father.'" "But in all the cases which have filled Westminster Hall with the most complicated considerations," continued Mr. Erskine, "the lunatics and other insane persons who have been the subjects of them, have not only had memory, in my sense of the expression, *they have not only had the most perfect knowledge and recollection of all the relations they stood in towards others*, *and of the acts and circumstances of their lives, but have in general been remarkable for subtlety and acuteness.* Defects in their reasonings have seldom been traceable—the disease consisting in the delusive sources of thought; all their deductions within the scope of their malady being founded upon the immovable assumption of matters as *realities*, either without any foundation whatsoever, or so distorted and disfigured by fancy, as to be almost nearly the same thing as their creation. It is true, indeed, that in some—perhaps in many cases—the human mind is stormed in its citadel and laid prostrate under the stroke of phrensy; these unhappy sufferers, however, are not so much considered by physicians maniacs, as to be in a state of delirium, from fever. There, indeed, all the ideas are overwhelmed, for reason is not merely disturbed, but driven wholly from the seat. Such unhappy patients are unconscious, therefore, except at short intervals, even of external objects; or are at least wholly incapable of considering their relations. Such persons, and such persons alone (except idiots) are wholly deprived of their un derstanding, in the Attorney General's seeming sense of that expression. But these cases are not only extremely rare, but never can become the subjects of judicial difficulty. *In other cases reason is not driven from her seat, but distraction sits down upon it along with her, holds her trembling upon it, and frightens her from her propriety.*"*

This is truthfully, powerfully, and eloquently enforced; but still, persons who are not conversant with the phenomena of insanity, find it difficult to recognise that *état mixte*, which has been so well described by Moreau as a form of insanity, in which reason appears to co-exist with madness,† a state which Shakespeare has so admirably portrayed, both in Hamlet and in King Lear.

"O matter and impertinency mixed,
*Reason in* MADNESS."
*Lear*, Act iv., sc. vi.

That sagacious and enlightened lawyer, Erskine, unreservedly admitted that "*insane persons often reason with a subtlety which puts in the shade the ordinary conception of mankind.*" But he argued, although these conclusions may be just, and frequently performed—"The premises from which they reason, when within the range of their malady, are uniformly false—not false from any defect of knowledge or judgment, but because *a delusive image, the inseparable companion of real insanity, is thrust* upon the subjugated understanding, incapable of resistance, because unconscious of attack." He therefore was led into the error of contending that "delusion," unaccompanied by frenzy or raving madness, should be regarded as the true test of insanity. This test was sanctioned by the authority of Sir John Nicholl, who in the case of Dew *v.* Clark, observed—"The true criterion, the true test of the absence or presence of insanity, I take to be the absence or presence of what, used in a certain sense, is comprisable in a single term, namely, delusion." Furthermore, the same learned judge added, "in the absence of anything in the nature of delusion, understood as above, the supposed lunatic is, in my judgment, not properly or essentially insane." The fallacy of this test was, however, aftewards conceded by Mr. Erskine himself, who, in defending a young woman indicted for murder, and acquitted on the ground of insanity, fully admitted that she did not labour under any delusion whatever. "The facts and circumstances which overpowered her understanding," he observes, "were strictly true: She was cast off by a Mr. Errington, with whom she had lived, and his marrying, or taking under his

* State Trials, vol. xxvii. Trial of James Hadfield for High Treason, p. 1313.

† Psychological Journal, 'Article "Mixed Insanity—Reason and Madness," vol. iii., p. 490.

protection, another woman, excited her grief and jealousy to such a pitch, that she could no longer control her actions. She accordingly, having procured a pistol, deliberately went to his house, where she shot him. She did not," he repeated, "act under a delusion that he had deserted her when he had not done so, but she took revenge upon him for his actual desertion of her." Every person who has made the study of insanity a speciality, now knows, that the disease may exist without any fixed or permanent delusion; although, when such delusions do exist, they are to be considered, according to their nature, as evidence of a state of mental derangement.

The next test of insanity—which was referred to at the trial of Arnold and of Bellingham, and which, indeed, has more frequently been appealed to than any other, from the time of Lord Chief Justice Hale down to the late decision of the twelve judges, in answer to the queries suggested by the trial of McNaughten,—affects the powers of moral discernment. Had the person, at the time of committing the offence, the knowledge of good and evil? was he capable of distinguishing right from wrong? In the case of Bellingham, the Attorney General (Sir Vicary Gibbs) declared, "upon the authority of the established law, in all times, which law has never been questioned, that although a man be incapable of conducting his own affairs, he may still be answerable for his criminal acts, if he possess a mind capable of distinguishing right from wrong." In his charge to the jury, Lord Mansfield—before whom the case was tried—reiterated the same view. "The single question," he said was, "whether, when he committed the offence charged upon him, he had sufficient understanding to distinguish good from evil, right from wrong; and that murder was a crime, not only against the law of God, but against the law of the country." In his work on Crimes and Misdemeanours, Russell adopted this doctrine; and Ray, in his Medical Jurisprudence of Insanity, points to that fact as indicating the little progress made in this department of science. "This opinion," he observes, "was delivered scarcely a dozen years after the absurdity of its principles had been so happily exposed, in a few words, by Mr. Erskine, on the trial of Hadfield. What a comment on the progress of improvement in the Medical Jurisprudence of Insanity!"* It is curious to find that, notwithstanding the light that has been thrown upon different species of insanity—especially upon moral insanity, homicidal insanity, and impulsive—by the highest authorities in his department of medical science, that the twelve judges should still have adhered to what we should consider an obsolete dogma. They declare that "the jury ought, in all cases, to be told

* A Treatise on the Medical Jurisprudence of Insanity. By J. Ray, M.D. With an Introductory Essay, by D. Spellan, M.D. London: 1839. Pp. 29.

that every man should be considered of sane mind until the contrary be clearly proved in evidence. That before a plea of insanity should be allowed, undoubted evidence ought to be adduced that the accused was of unsound mind, and that, at the time he committed the act, he was not conscious of right or wrong. Every person was supposed to know what the law was, and therefore nothing could justify a wrong act, except it was clearly proved that the party did not know right from wrong. If that was not satisfactorily proved, the accused was liable to punishment. If the delusion under which a person laboured were only partial, the party accused was equally liable with a person of sane mind." Such is the present state of the law; yet there is no fact better established than that insane persons, criminal lunatics in particular, are frequently perfectly conscious of the distinction between good and evil, right and wrong, and even the consequences which will attend their committing certain acts; but, notwithstanding all this, they are unable, from their state of mental infirmity or aberration, to control their morbid propensities.

This brief retrospect, showing how improgressive are the principles which govern our learned judges in dealing with the plea of insanity in criminal cases, and what little sympathy and coincidence exists between legal and medical views on the subject, we have thought a befitting introduction to our notice of Dr. Mayo's Cronian Lectures on Medical Testimony and Evidence in cases of Lunacy, and a *brochure* recently published by Mr. Knaggs, entitled "Unsoundness of Mind considered in Relation to the Question of Responsibility for Criminal Acts." The report of the lectures of Dr. Mayo, in the medical journal before us, we presume to be considerably curtailed; they are three in number. In the first, he discusses the subject of insanity in its relation to medical proof under its essential element, delirium, as indicated by inconsecutive, incoherent trains of thought, and by certain delusions over which the patient has no control; in the second, he dwells upon the destructive orgasm or tendency in its relation to moral insanity especially; and in the third, he considers idiocy in its relation to civil actions, and the distinctions which may be drawn between unsoundness of mind and insanity. The work of Mr. Knagg is condensed into seven chapters.—I. The Introduction.—II. On Mind.—III. Sound and Unsound.—IV. Punishment in Reference to Crime and Lunacy.—V. Unsound Mind as a Responsible Condition.—VI. Unsound Mind as an Irresponsible Condition.—VII. The concluding Chapter containing "practical suggestions." Upon the *questio vexata*, what should be esteemed the true test of insanity as regards the responsibility or non-responsibility of the criminal lunatic, neither author appears able to lay down any practical rule for our guidance. Dr. Mayo, indeed, suggests that insane persons may be divided into two classes, the responsible and the irresponsible, and he suggests that "the responsible insane should undergo some lower degree of punishment than that inflicted on similar delinquents being of unsound mind." But a man, it is obvious, must, in the eye of the law, be held either sane or insane; if sane, the usual sentence upon conviction is, of course, passed: but if insane, why should the unfortunate person be subjected to any degree of secondary punishment? "The position of many persons under capital charges," continues Dr. Mayo, "is at present anomalous. They are acquitted in defiance of the law, as laid down by the judges respecting McNaughten's case, because the punishment appertaining to the offence would be too severe; and then, instead of being consigned to confinement in a gaol as a secondary punishment, they are consigned to it in an asylum as a plea simply of detention. This becomes a scene of severe virtual punishment to some of them; of gratification to vanity, and idleness to others; those, meanwhile, to whom it is a grievance, as they do not regard it in the light of a punishment, derive from it none of the preventive effects of punishment or future conduct; while the public, for the same reason, find it equally unproductive of good, as an example to persons of actually diseased mind, or to that large class of persons who are drifting into disease under uncontrolled eccentricity." We confess it is new to us that, in any case, the plea of insanity has ever been admitted because "the punishment appertaining to any particular offence would be too severe;" and we certainly should object to asylums, which ought to be regarded as hospitals for the cure of mental disease, being converted into supernumerary gaols; in all the cases that ever have occurred, when the plea of insanity has been raised, the only question for the judge and jury to decide, has been whether the accused was sane or insane; responsible or irresponsible for his actions. And the whole difficulty hinges upon how this is to be determined? It is proposed by Dr. Mayo, that the alleged criminal lunatic should be examined as to his state of mind in the presence of the judge and jury, which would, he assumes, "facilitate an understanding between law and medicine in the most Protean form the most untractable subject of investigation which is brought before either: and in doing this, give full weight and value to the deductive reasoning of lawyers, while we support the claim of the medical profession to aid them in the work of decision or inquiry." In criminal cases, a grand jury has no authority by law to ignore a bill of murder on the ground of insanity, but if a man be found insane upon arraignment, he is not tried, because he is incapable of adducing evidence in self-defence; if, however, the trial proceeds, counsel does take advantage of the presence of the prisoner at the bar, to point out, as Dr. Mayo suggests, any personal peculiarity or appearance that may support the plea of insanity. Thus, upon the trial of Hadfield, the attention of the jury was called to the wounds of the head which he had received while serving in the army. "When the court," said Mr. Erskine, "put the prisoner under my protection, I thought it my duty to bring Mr. Cline to inspect him in Newgate; and it will appear by the evidence of that excellent and conscientious person, who is known to be one of the first anatomists in the world, that from this wound one of two things must have happened; either that, by the immediate operation of surgery, the displaced part of the skull must have been taken away, or been forced inward on the brain. The second stroke also speaks for itself; you may now see its effects;" here Mr. Erskine placed his hand on the head of the prisoner, showing to the court the injuries he described, and then added, "he was cut across all the nerves which give sensibility and animation to the body, and his head hung down almost dissevered, until, by the aid of surgery, it was placed in the position you now see it; but thus almost destroyed, he still recollected his duty, and continued to maintain the glory of his country, when a sword divided the membrane of the neck where it terminates in the head; yet he still kept his place, though his helmet had been thrown off by the blow which I secondly described, when, by another sword, he was cut into the very brain—you may now see it uncovered." The learned counsel added, "*There* the disease is, from its very nature, incurable; and so when a man like the prisoner has become insane from violence to the brain, which permanently affects its structure, however such a man may appear occasionally to others, his disease is immovable; and if the prisoner, therefore, were to live a thousand years, he never could recover from the consequence of that day." There can be no doubt that the presence of the unfortunate man in the midst of this scene, must have strikingly affected the whole court; but we are not prepared to affirm that it would have been expedient for him to have been personally examined and cross-questioned at the bar by any medical men. This practice, if introduced into our courts, whether civil or criminal, would lead to very painful exhibitions; and the jury, being unacquainted professionally with the phenomena of insanity, would be little able to appreciate the value of the evidence. A sane man ingeniously cross-examined by medical men, assuming the functions of barristers, might be made to appear in open court insane enough; and, on the contrary, a lunatic may summon up sufficient energy of mind to answer questions with marvellous lucidity, whereby the jury again would be misled. But the most fatal objection to adopting the suggestion of Dr. Mayo is, that no man upon trial for his life is bound to convict himself: the humanity of the English law protects him as much as possible from being a witness, under any criminal indictment, against himself. Far more humane and efficient is the present system. The physician who has made the study of insanity a speciality, and who has attained eminence in this branch of the profession, is desired to visit the alleged lunatic. This he may do repeatedly; and when he has satisfied himself as to his state of mind, his evidence is delivered in court. This is, in our estimation, a far better and more satisfactory mode of proceeding than the one suggested by Dr. Mayo; we have more frequent opportunities of testing the sanity of the prisoner, and discharging more satisfactorily to our own mind one of the gravest responsibilities which can devolve upon us.

In the lectures before us, Dr. Mayo tells us that he has accepted the terms which are laid down by Acts of Parliament as expressing the conditions on which abnormal states of mind are imputable; and with respect to their meaning, while he does not consider them virtually synonymous, or as having the same force, he has endeavoured to adopt that which the law may be presumed to intend, and which expresses real not verbal differences. It is to be regretted, as Dr. Mayo appears himself to intimate, that he did not take a larger basis for his division of insanity, and one more consonant with what he designates "the entire pathology of the mind;" he would not, in that case, have repudiated, as he appears to do, the forms of "impulsive," "instinctive," and "moral insanity."

"With respect to eccentricity and atrocity of vice," says Dr. Mayo, "I may observe, that the theory of either moral or impulsive insanity is liable, for anything that Dr. Prichard has suggested, to occasion the sudden outbreaks of the brutal character—a character under rapid devlopment at present in the country—to find refuge under this plea. Such was the application of it which some years ago protected the honourable Mr. Tucket from the penal consequences of a great crime. That gentleman put to death by a pistol-shot the marker of a shooting gallery. The act was sudden, and there was no appearance of motive; but it was not performed under any semblance of delirium. Mr. Tucket was eccentric; and he was *blazé*. He fancied that he desired to be hanged: at the gallows he would probably have thought differently, and he was reckless and brutal enough to give himself a chance of this fate at the expense of the life of a fellow-creature. I have noticed him since in the criminal department of Bethlem, *insouciant*, and indifferent enough, but certainly not insane in any sense of the word that would not entirely disintegrate its meaning; neither, when we proceed to consider the sense which the law inteuds to give to the expression of the certificate, 'unsoundness,' shall we find this epithet at all more appropriate to Mr. Tucket's case, which was simply one of brutal recklessness."

Now, with great deference to Dr. Mayo, whose opinions we respect, it appears to us that no man in his sound senses would commit a murder for the sake of being hanged; and from our recollection of this case, it came clearly under the head of homicidal and impulsive mania. There may have existed in the mind of this unfortunate person a desire to commit murder, and a desire to be hanged; for it is notorious that homicidal is very frequently, if not generally, complicated with suicidal mania. Many such cases are on record. We also, contrary to the opinion of Dr. Mayo, believe that the court was right in deciding that Captain Johnson, who, cruelly wounded and murdered, upon several successive days, many of his ship's crew, was also insane when he committed those outrageous acts; and it seems to us to militate as an argument against Dr. Mayo's suggestion of the alleged lunatic being examined in the presence of the judge and jury, that if, as he conjectures, such a course had been adopted in the case of this man, instead of being treated as a lunatic, he would not have escaped punishment, in other words, he would have been found guilty and hanged; but no "shades of psychological distinctions" can, in our opinion, justify the infliction even of secondary punishment upon a lunatic. He must be treated as a person who is either sane or insane, as being morally responsible for his actions or morally irresponsible: there can be no intermediate condition. It appears that Dr. Mayo would not allow persons guilty, like Tucket and Johnson, of "brutal recklessness" to escape under the plea of impulsive or moral insanity; and he suggests, "whether a state of the human mind is not conceivable which shall be distinguished entirely from insanity as implying no delirium,—from unsoundness as implying no incapacity for the conduct of person and property,—and which shall bear the same relation to the moral sense as idiocy bears to the intellectual sense, involving an entire absence or imperfect development of the former, as idiocy does of the latter? A complete practical division of, or system of abnormal mind, would comprehend such a head, and the distinction would involve none of the mischief which I have imputed to the doctrines of moral insanity. That mischief is contained in one short expression—impunity afforded to crime. No such impunity is implied in the hypothesis which avowedly represents the abnormal state as a mode of wickedness consisting *in the non-development or absence of the moral sense.* For, from being thus constituted to remove the fear of punishment, would be to denude him of the sole preventive of crime afforded him by Providence." This hypothesis is put very ingeniously by Dr. Mayo; but may it not fairly be argued, that to punish a man for a connatural defect of mind would be as unjust as it would be to punish an idiot for being incapable of using the reflective faculties? If there be, in the original constitution of the mind, an entire absence of so important an element as the moral sense, which is the true arbitrator between right and wrong, and the basis, according to many ethical writers, of all good and virtuous actions, such a person is as much to be pitied as any congenital cripple, who might as well be punished for not using limbs which Nature has, contrary to her usual custom, denied to him. It is clear that a person so afflicted ought to be protected; for this defect in his mental organization takes from him the power of distinguishing right from wrong, and reasoning correctly on the consequences of his actions. Hence he may become predisposed to commit those motiveless and impulsive outrages which Pinel, Esquirol, and Prichard have very properly described as arising from that species of the disease designated moral insanity. We have not time to pursue this interesting subject further; the existence or non-existence of a moral sense, as a distinct and original faculty of the human mind, would lead to a disquisition upon the views of Cudworth, Locke, Hutcheson, Price, and other ethical authors, which would far exceed our limits. Suffice to say, that if we recognise, as Dr. Mayo seems to do, the existence of a moral sense in the natural constitution of the human mind, its non-development or absence would subject the mind to a state of irrationality which has a strong claim upon our sympathies, and which should demand for a person so afflicted the same consideration as if he became insane from any other cause.

In Dr. Mayo's third lecture, he discusses the meaning of the expression, "unsoundness of mind," as used in our medical certificates of lunacy, in contradistinction to insanity and idiocy. "Unsoundness of Mind," he tells us, "is the term which applies to a person of whom neither insane delusion, nor inconsecutiveness, nor incoherency can be predicated—but who may nevertheless be brought before a physician as requiring precautions in reference to the management of his property and person. The man in question is not in his dotage—he talks with sufficient fluency and without anything remarkable in the sequence of his thoughts. But on every subject of business his mind goes into a state of confusion, of which he is not conscious. He is unable to appreciate value, and though fond of property, will purchase and part with it at absurd prices. He can believe anything that is told him, however improbable, and if he takes a dislike can invent or believe any fiction that falls in with his feelings of resentment. I may add, that his conversation being on the whole continuous and coherent, there is never flighty character in it, and often some deficiency of articulation." We recognise readily the truthfulness of this portrait, which introduces to us a class of patients whom it is extremely difficult to deal with—requiring, as they do, protection rather than confinement; a species of surveillance which shall not remind them that they are considered insane persons—but which shall at the same time be sufficiently stringent to prevent their injuring themselves or others. The first example which Dr. Mayo has selected to illustrate this form of insanity—is that that of the late Mrs. Cumming—but as we propose at some future time discussing at some length this important case in all its medico-psychological relations, we abstain for the present making any remarks upon the interpretation which Dr. Mayo gives to the incidents he has referred to.

The same theoretical difficulties which appear to have occurred to Dr. Mayo in the course of his Croonian Lectures, have obviously embarrassed Mr. Knagg's speculations, who has, we fear, not succeeded in throwing any new light on the subject—nor could this be expected, as he appears to have argued only within the same circle as his predecessors. He, too, refers to the cases of Hadfield, McNaughten, Laurence, Touchet, &c., and finds himself equally at a loss to determine what test should be adopted to justify a criminal lunatic being released from responsibility. The decision of the twelve Judges—that "nothing could justify a wrong act, except it was clearly proved that the party did not know wright from wrong," Mr. Knaggs repudiates. "A full consciousness," he observes, "of the illegality of wrongfulness of the act may exist in a man's mind, and yet he may be fairly acquitted on the ground of insanity; thus the incendiary Martin admitted, that he knew he was doing wrong according to the law of man, when he set fire to York Cathedral; he was conscious that the act was illegal, but said he had the command of God to do it." Moreover, "not unfrequently do we find existing in the lunatic criminal, not only a consciousness of right and wrong, but even a knowledge of the consequences of the act; and strange to say, perhaps this latter perception constitutes the only motive for the commission of the deed. Thus, in the case of Hadfield, he knew that in firing at the king he was doing what was contrary to law, and that the punishment of death was attached to the crime of assassination, but the motive for the crime was that he might be put to death by others; he would not take his own life." Many years ago, we ourselves went over this ground, and as a proof, that in many cases of insanity, the patient appears perfectly competent to perform a correct process of reasoning, and is fully aware, not only of the distinction between right and wrong, but of his legal responsibility; we cited the two following anecdotes:—An intriguing, unruly, vicious madman, was detected with a piece of iron, which he had contrived to shape like a dagger; into this iron he firmly fixed a handle. The weapon was taken away from him. He immediately became excessively abusive, and was placed under restraint. After this, he was more violent, and uttered the most revolting imprecations. In a fit of fury, he exclaimed to the attendant, "I'll murder you yet; I am a madman, and they cannot hang me for it." When Martin set fire to York Minster, a conversation took place among the inmates of a neighbouring lunatic asylum relating to this circumstance. The question discussed was whether Martin would suffer the extreme penalty of the law for the crime. Various were the opinions expressed. In the midst of the conversation, one patient, as mad as the rest, exclaimed " He (Martin) will not he hanged,—of course he will escape." For what reason?" asked several voices. " *They cannot hang him,*" replied the lunatic, " *because he is mad—he is one of ourselves.*"* Any person who has had the charge of insane patients, and lived among them, whether in a public or private asylum, will bear testimony to many of them conducting themselves very rationally; nay, they will often converse upon the subject of their own malady, and ridicule their own insane actions. Not one of his tests, therefore, referred to at the commencement of this article, and propounded by the most learned of our judges, has been found to hold good; indeed it is well observed by Dr. Mayo, in one of the lectures before us, that there is a remarkable discrepancy between the theory and practice of the law. " The position of many persons under capital charges," observes the doctor, " is at present anomalous; they are even acquitted in defiance of the law." Thus it would appear—and we fully concur with Mr. Knaggs in this remark—" That any attempt by fixed rules, either legal or medical, to distinguish between those mental conditions which should be accountable, and those which should not, must either fail in its application or be productive of evil." With this admission, we are somewhat surprised to find Mr. Knaggs suggest a new test, which is quite as unavailable as any other. " The best test of the responsibility of the criminal on the plea of insanity is, not whether he be conscious of right and wrong,—or have a knowledge of the consequences of his act,—but whether he be capable of controlling his actions—not alone in homicidal cases, but wherever the plea is raised."—(pp. 69—77). Were it possible to solve this problem, there would be an end of the difficulty; but Mr. Knaggs himself admits that the crime may be proved; but—we quote his own words,—" We have no means of ascertaining the actual state of mind of the person at the time of his committing the act."—(p. 76.) What then becomes of the proposed test?

We have compared attentively Chapter IV., entitled, "Unsound Mind, as a Responsible Condition," with Chapter V. " Unsound Mind as an Irresponsible Condinion." But we confess we cannot discover

* The Plea of Insanity in Criminal Cases. By Forbes Winslow, M.D, London: 1843.

any criterion for determining the amount of mental unsoundness which shall place one criminal lunatic in a responsible—another in an irresponsible condition. We return, as we have just observed in reference to Dr. Mayo's proposal for inflicting secondary punishments in such cases, to the pith of the question. Was the man arraigned upon a criminal charge, when he committed the alleged offence, in a sound or unsound state of mind? Was he sane or insane? We cannot, upon a truely conjectural theory, allow assumed "shades of psychological distinctions"—as Dr. Mayo expresses it—to exculpate one lunatic and exonerate another. Who shall presume to determine where, in a mind confessedly unsound, responsibility shall end, and irresponsibility begin? We may invent supposititious cases in support of any hypothesis, but when we come to deal with the stern realities which are brought forward in our criminal courts, when we are put into the witness-box as physicians, conversant with the general and special phenomena of insanity, and are called upon to state the views we entertain and the conclusions we have arrived at from experience, these speculative and shadowy distinctions disappear and become merged in the general fact, that when the accused committed the crime libelled against him he was in a state of unsound mind. This to us is sufficient; we cannot probe deeper. When the life of a human being is at stake we will not split straws respecting the abnormal conditions of mental faculties, the specific range of which we know little of in health, and less of in disease. We, therefore, after carefully reviewing Mr. Knagg's arguments, return to the point from which we started, which may be thus expressed:—No medico-psychological rule or formula can be laid down for determining when a man, already of unsound mind, loses the power of controlling his actions: urged by an insane impulse, he may at any moment, like Touchet or McNaughten, commit an outrage; but it is impossible for us to determine at what precise moment the insane impulse either took possession of his mind or became irresistible; we can adjudicate only upon overt acts. The all-seeing eye of Heaven can alone penetrate into the secrets of the human heart. We cannot watch the course of a man's silent thoughts, nor can we weigh the evil passions which may be slumbering in his breast; but if, as medical jurists, we believe him to be of unsound mind, assuredly the plea of his insanity should be allowed to prevail in his defence.

We know from our own experience how perplexing it is in some cases to draw a satisfactory diagnosis, and how careful we ought to be in weighing every collateral circumstance which can throw any light upon the motives which may have actuated a criminal lunatic. So conscious, indeed, does Mr. Knaggs appear to be of the difficulty of determining the question of responsibility or irresponsibility, that in the chapter of "Practical Suggestions," he proposes that the opinion of a single physician, however emiment in the profession, should not be relied upon; but that a jury of medical practitioners, selected from amongst those who have had experience in the observation or treatment of the insane, should be empannelled, and that upon their verdict the fate of the prisoner should depend. But this verdict, continues Mr. Knaggs, should not be founded upon the fact that the prisoner is of unsound mind, "but upon the condition of a sufficient degree of unsoundness in their judgment being present to constitute a plea as to irresponsibility. Such a jury should consist of three or more, with a foreman, also medical, to collect their verdict; then supposing, in the course of an ordinary trial, that the plea of insanity was raised, the decision upon this ground would rest with the empannelled medical jury, and in the event of their rejecting the plea upon hearing the evidence, the common jury would proceed as usual to their verdict; but if the panel was of opinion that the evidence of unsoundness was sufficient to constitute irresponsibility, the decision should be held to be final, and a verdict of 'not guilty,' on the ground of insanity, recorded by the judge."—(p. 84.) This appears to be a very ingenious suggestion, albeit, somewhat utopian; but if one experienced physician cannot solve the psychological problem upon which their verdict is to depend, we are afraid the wisdom of three would be equally unsuccessful: nay, we cannot understand by what *scale* these *degrees* of mental unsoundness are to be measured; therefore we should infinitely prefer the verdict, resting as it at present does, upon the general fact,—whether the prisoner was or was not of unsound mind or insane when he committed the act? This is the question for the judge and the jury to determine; and instead of a *medical* jury being empannelled to express their opinion in the form of a verdict—the plan at present adopted—that of calling in the evidence of the most eminent men in this department of the profession is by far the best. There may occasionally be an unseemly difference and collision of opinions among specialists, but even this tends to the elucidation of the actual truth; and the prisoner, if insane, has an advantage which he would not possess were his fate dependent upon the verdict of a small compact jury of three or four medical men. It is clearly more just—more for the benefit of the prisoner, and more satisfactory to the public—that the opinions of many physicians of eminence should be received in evidence.

We must, however, now conclude. The Croonian Lectures, by Dr. Mayo, will, we hope, be published in a more complete form; we have selected passages for criticism rather than for praise, or we might have given many interesting extracts which would show that these lectures will be a valuable contribution to the literature of medical jurisprudence. Insanity ever has, and ever will be a perplexing subject even to the most profound psychologists. The most eminent authors have failed to give any satisfactory definition of the disease. It is a popular notion that every man is a little mad, and we confess we have been much amused in reading the following description of the great lexicographer, Dr. Johnson, whose eccentricities grouped together are exceedingly striking. The portrait is admirably drawn:—

"There was" (says Mr. Knaggs) "an old man well known in London during the last century, who was of an ungainly appearance, and subject to occasional attacks of hereditary melancholy; so inconsistent was he in his habits that sometimes he practised great abstemiousness, and at other times devoured huge meals with brutish slovenliness and voracity; sometimes he would persist in drinking nothing stronger than water, but occasionally drank wine by tumblers full: his income was far from large, and not of a certain amount, yet he kept a set of old men and women about his house, whose bickerings and disagreements now and then drove him out of doors; he was in general very loquacious, but had been known to sit in company and drink a dozen cups of tea without speaking a syllable; when not engaged discoursing, it was his custom to keep muttering to himself; in walking, he performed strange gesticulations with his limbs, and would not go in at a door unless he could effect his entry in a certain preconceived number of steps, and so as to introduce himself on a particular foot, turning back and recommencing until he succeeded as he desired; there was a row of posts near his house which he would not pass without touching singly, and if he had omitted one in the series, he retraced his steps to remedy the neglect; he hoarded up orange skins for some mysterious purpose which he would never divulge; he suffered remorse of conscience for having taken milk in his coffee on Good Friday; he believed in ghosts and went ghost hunting in Cock-lane; and he maintained he had heard his mother calling upon him by name in the other world. Yet Dr. Johnson was so far from insane, that his judgment commanded respect and admiration everywhere, and, by the common consent of eminent contemporaries, he was the most vigorous thinker and the greatest sage of his time." (p. 46.)

We regret we have not space for further extracts. We have found Mr. Knagg's work extremely interesting; every page may be taken as a text for a running commentary, and ere long we may have occasion again to return to it.

